# Surgery as a greedy job: The valorization of the worker's professionalism for women in surgery

**DOI:** 10.1016/j.sopen.2026.02.002

**Published:** 2026-03-04

**Authors:** Maria Irene Bellini, Pasquale Passalacqua

**Affiliations:** a Department of Surgery, Sapienza University, Rome, Italy; b Department of Law, Tor Vergata University, Rome, Italy

**Keywords:** Surgery, Greedy job, Women in surgery, Discrimination

## Abstract

**Introduction:**

Greedy job is referred to a condition in which companies and/or institutions require employees to work beyond agreed-upon working hours or beyond agreed standards. With the current shortage of surgeons and increased number of women entering a surgical career, it is fundamental to consider their needs, distress, and motivation factors to implement the surgical workforce.

**Methods:**

A systematic search was performed to review literature addressing working hours disrespect and greedy jobs in surgery, aiming to report the current evidence with regards to the different impact on general surgeons.

**Results:**

Greedy job in surgery is common and its consequences are predominantly affecting female gender and younger generation. Prolonged working hours were associated with burnout incidence, reduced time for research and eventually jeopardizing education. Furthermore, job satisfaction, earning and career progression discrepancy between genders, as well as the risk for health and wellness with secondary effects on parenthood and childcare were predominantly hindering women surgeons.

**Conclusions:**

Sustainable and flexible working hours could represent a valuable opportunity to attract future generations into surgery. This is particularly important for the recruitment of women, who historically have more frequently faced the negative consequences of greedy jobs. Technology might be in the future of aid to overcome space and time constraints, always with the goal to protect rest time, personal life, and mental and physical health, from a perspective we might call “anti-greed”.

## Introduction

Discrimination against women pursuing careers in the surgical field has deep historical roots, dating back to the very beginnings of the profession. This prejudice is starkly illustrated by figures like Sir James Paget, a prominent 19th-century British surgeon, who cautioned against women's entry into surgery could lead to the “disorganization of society,” fundamentally reflecting anxieties that challenging and defying traditional, defined gender roles would affect the established social order. Strikingly, despite over a century and a half of societal and professional evolution, this inherent bias remains a palpable reality: a survey conducted as recently as 2020 revealed in fact that only 67% of male surgeons expressed confidence in the professional competence of their female colleagues [1]. This lingering skepticism is often rationalized through a lens of gender-oriented societal norms, which disproportionately focus on women's assumed familial responsibilities and the perceived lifestyle implications these responsibilities have on their professional commitment and availability. This perspective subtly, yet powerfully, suggests that women's professional capabilities are inherently compromised by their roles outside of the operating room, perpetuating a cycle of discrimination that has plagued the surgical field since its inception.

The persistence of such attitudes highlights the systemic and cultural barriers that continue to impede true gender equality within surgery [2], therefore, as a professional field, it remains largely male-dominated, well beyond a simple pipeline issue.

Although such opposition, remarkable efforts have been paving the way to change, and in fact currently, women represent >50% of the medical workforce in many Western Countries, especially if we consider professionals aged <50 years [3]. However, despite these encouraging data, substantial gender gap persist, in a profession where restriction for duty hours have been controversial, including the downsides commonly hindered by “greedy” jobs.

As per 2023 Nobel Prize Claudia Goldin definition [4], greedy jobs identify situations in which companies and/or institutions require employees to work beyond agreed-upon hours or beyond certain standards. Employees of both sexes might face difficulties in meeting these demands, resulting in a conflict between work and life balance. In particular, it has been recognized as a possible cause of discrimination in accessing higher-skilled and more remunerated positions, especially for women.

These phenomena fall within the metaphor of the so-called “glass ceiling”, which refers to the socio-cultural barriers [5], usually invisible and insurmountable, that prevent minorities from opportunities for professional and career development, as well as the “sticky floor”, a discriminatory employment model that keeps the same individuals at the lower rungs of the job ladder, with limited mobility.

The aim of this review is to explore the current evidence in literature regarding women working in general surgery according to the implications of a “greedy job” definition, highlighting the discriminations implied and aiming at possible system developments for the valorization of each worker's professionalism.

## Methods

**Fig. 1:**
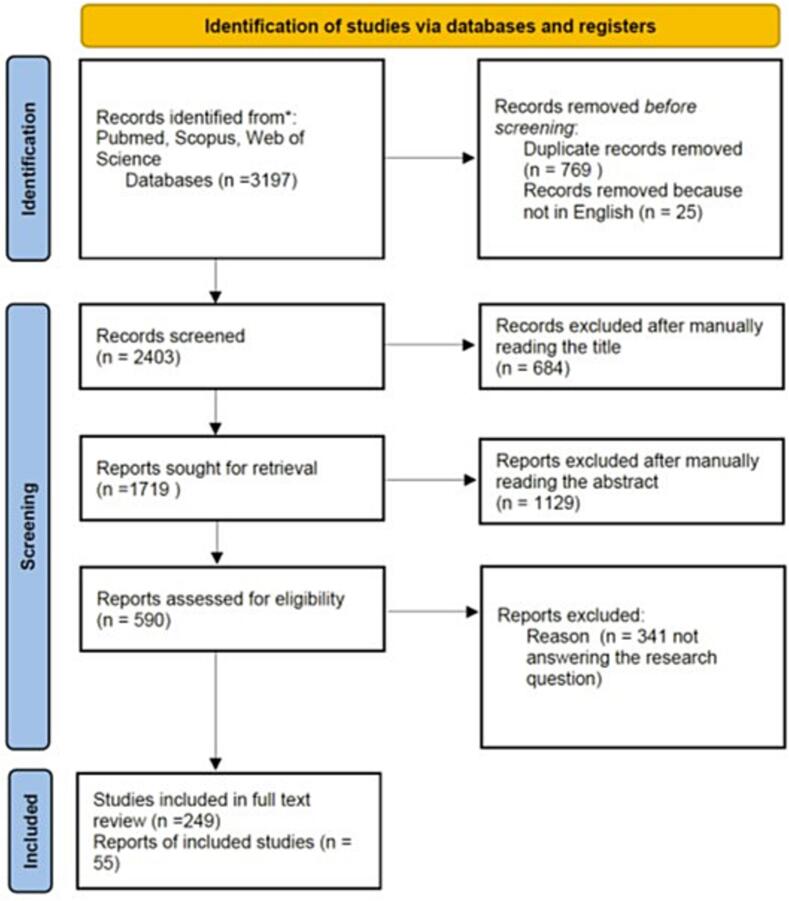
PRISMA flow diagram.

A systematic search was performed within the PubMed, Scopus, and Web of Science electronic databases. Only original articles in English published from 2000 to date, and reporting on the work condition identifiable as greedy job, as previously described [4], were considered. Search terms included combinations of the keywords “greedy work”, “women in surgery” and “working hours”. The question we aimed to answer was “What are the implications of prolonged working hours for women surgeons?” Focus was posed on conditions associated with less than full time work, flexibility, career and salary progression and work-life conflicts, seen as mainly affecting women. We included all the articles written in English, with no zone restriction and reporting on general surgery and its subspecialties. Papers written in languages other than English, focusing on COVID-19 and disciplines other than general surgery were excluded from the analysis. We then narratively synthesized the themes extracted and summarized the findings in Table 1. The PRISMA diagram for the search is reported in Fig. 1.

**Table 1:** Study characteristics, aims, and main findings.

Author	Year	Country	Method	Objective	Percentage of Female Participants (Female/Total Participants)	Main findings
Antiel et al.	2013	USA	Survey	Impact of duty hour regulations on QoL and burnout on surgical interns	50/156 (32)	Satisfaction from being a surgeon was high, but 1 in 7 considered giving up their career
Arora et al.	2008	USA	Survey	Program Directors views regarding duty hours	(10)	A group of PDs reported having no reduction in clinical duties, no protected time, and minimal salary compensation
Ashrafi et al.	2024	Pakistan	Survey	To assess the optimum working hours for surgical interns and supervisors	27/33 interns (82)	The majority of residents and supervisors recommended reduced working hours
Balch et al.	2010	International	Survey	Assessments of burnout and QoL	1037/7757 (15)	Burnout and other measures of surgeon distress correlate directly with increasing work hours and nights on call
Baldwin et al.	2009	USA	Survey	To assess activities residents engage in, and how much time per week they spend in each activity	378/617 (60)	Activities outside of work and sleep hours correlate highly with residents' mood, learning, and satisfaction
Barden et al.	2002	USA	Survey	How the surgical residency training program at New York Hospital was modified to comply with Code 405 mandates	N/A	Reduction in resident work hours has salutary effects on perception of quality of life and basic education for surgical residents
Barger et al.	2006	USA	Survey	To address the impact of extended-duration work shifts on patient safety in a large and more diverse population of interns	(53)	Extended-duration work shifts were associated with an increased risk of significant medical errors, adverse events, and attentional failures for fatigue
Bendorf et al.	2010	USA	Survey	To evaluate working hours issues and identify factors impacting breast surgeon incomes and job satisfaction	(41)	Significantly higher job satisfaction scores among dedicated breast surgeons, despite the lower salaries
Bennett et al.	2017	USA	Survey	To assess underreporting duty hours among surgical residents	466/1003 (46)	Female respondents more often cited exceeding duty hours owing to the guilt about leaving the hospital
Bessen et al.	2025	USA	Retrospective analysis	Evaluation of EHR use by surgeons	30/91 (33)	Women surgeons had a greater EHR burden than men, that might contribute to gender gap in career advancement and burnout
Bilimoria et al.	2016	USA	Survey	To assess adherence to duty hour requirements in the Standard Policy arm and examine how often and why duty hour flexibility was used in the Flexible Policy arm (FIRST Trial)	1501/3795 (40)	There were differences in duty hours worked by residents in the Flexible vs Standard Policy arms of the FIRST trial, but it appeared that residents generally used the flexibility for patient care and educational opportunities selectively
Boerjan et al.	2010	Netherlands	Survey	To observe the influence of gender and number of working hours on health, social support and job-related autonomy	74/254 (29)	Social support by supervisors strongly predicted health; men and senior residents were healthier than women and junior residents, respectively, and a high level of support by colleagues predicted health in female residents
Brown et al.	2021	USA	Survey	To assess WLB and burnout for trauma surgeons	84/291 (29)	Females have higher burnout rate, with 43% being satisfied with WLB. Factors associated with a satisfying WLB were: lifestyle and fair compensation; midcareer, increased hours at work, decreased awake hours at home
Chokshi et al.	2009	USA	Survey	To explore the following 4 domains: demographics, time allotment, research activities, and effects of stressors	36/314 (12)	Female are more active in research than men, however, this involvement decreases as other profess-sional responsibilities increase
Coverdill et al.	2006	USA	Survey	How surgical residents and faculty assessed the first year of the Accreditation Council for Graduate Medical Education duty-hour restrictions	47/252 (19)	Residents, especially female residents, view the restrictions more favorably than faculty
Dahlke et al.	2018	USA	Survey	To assess differences in how male and female general surgery residents utilize duty-hour regulations and experience aspects of burnout and psychological well-being	2831/7395 (38)	Female more frequently stayed in the hospital >28 h or worked >80 h in a week and more frequently felt fatigued and burned out
Dholakia et al.	2025	New Zealand	Survey	To quantify the prevalence of burnout in surgical specialists and registrars at a tertiary center and identify contributory factors	67/110 (61)	Contributory factors were frustration with management, lack of resources and long working hours, with predominance towards fatigue and service provision over career progression among the registrar group
Dossa et al.	2019	Canada	Retrospective analysis	To determine whether male and female surgeons have similar earnings for each hour spent operating in a fee-for-service system	162/705 (23)	Even when equal hours are worked, female surgeons earn less than male surgeons and have fewer opportunities to perform the most lucrative surgical procedures
Dyrbye et al.	2011	USA	Survey	Burnout and career satisfaction	1043/7858 (13)	WHC appear to be a major contributor to surgeon burnout and are more common among women surgeons
Dyrbye et al.	2012	USA	Survey	To evaluate the relationship between WHC and the personal and professional characteristics of surgeons	879/6240 (14)	Hours worked per week, having children, sex, and work location or were independently associated with an increased risk for WHC
Elmore et al.	2016	USA	Survey	Burnout	289/664 (44)	69% surveyed met the criterion for burnout; of these 73% were women
Gifford et al.	2014	USA	Survey	To determine how often categorical general surgery residents seriously consider leaving residency	112/288 (39)	Women were more likely to continue to have serious thoughts of leaving as residency progressed for sleep deprivation and difficult interaction with faculty
Goitein et al.	2008	USA	Survey	To determine the effects of the resident WHL on the professional lives of faculty	77/282 (28)	Women were more than twice as likely as men to report a decrease in time for research or other academic pursuits because of WHLs and more than three times as likely to report an increased sense of responsibility for supervising patient care
Gray et al.	2019	USA	Survey	Comparison of responses between men and women to detect any differences in career goals, salary expectation, and perspectives towards salary negotiation at a resident level.	177/407 (44)	Overall career goals were similar for men and women; however, women had lower future salary expectations and a significantly more negative view of salary negotiation
Hoffmann et al.	2017	Canada and Switzerland	Survey	To compare 2 surgical training programs with the potential to identify opportu- nities for program enhancement	30/105 (29)	Although residents and consultants in both institutions fear negative influence of reduced working hours on the training program, this was not the case Canada's residents
Hughes et al.	2023	Canada and USA	Interviews	Factors influencing surgeons' wellbeing	8/17 (47)	Women may have experienced respect and recognition issues more often than men
Hughes et al.	2024	International	Survey	To utilize the concepts of stressors, satisfaction	72/ (32)	Paperwork and documentation were sources of frustration as well as problems with billing, coding and reimbursement
Irani et al.	2005	USA	Survey	To determine perceptions of the effects of the ACGME duty hour requirements	65/238 (27)	Residents report an improved QoL
Janczewski et al.	2024	USA	Survey	To assess factors associated with WLC and well-being	2253/5133 (44)	Female trainees were more likely to experience WLC, associated with career dissatisfaction, burnout, thoughts of attrition, and suicidality
Kang et al.	2015	Korea	Survey	To evaluate the level of occupational stress of Korean surgeons compared to other professions	100/621 (16)	Young age and female gender were found to be related to a high occupational stress scale
Kawase et al.	2024	Japan	Survey	To assess factors affecting marriage and pregnancy/childbirth	(100)	Age at the first childbirth is higher
Kevric et al.	2018	Australia	Survey	To provide contemporary data on the mental health of surgical trainees and identify risk factors relating to poorer mental health outcomes	34/83 (41)	Hours of overtime worked, particularly unpaid overtime, were strong predictors of a poorer score
Khorfan et al.	2020	USA	Survey	To investigate the long-term effect of flexible duty-hour policies on resident outcomes	1494/ (40)	Residents in flexible duty-hour programs reported significantly fewer lapses in continuity than standard policy residents
Kinslow et al.	2020	USA	Survey	To identify the prevalence and nuances of reported burnout in general surgery resident to better understand which factors contribute the greatest risk	39/81 (48)	Both female and community-affiliated residents were at increased risk of reporting suicidal ideation.
Kiyasu et al.	2024	Japan	Survey	To identify the problems trainees face during surgical training	194/758 (26)	Main identified issues related to long working hours and harassment
Kuerer et al.	2007	USA	Survey	Burnout and career satisfaction	108/548 (20)	Burnout was more common among respondents <50 years and women. Devoting <25% of time to research, lower physical QoL, and ≤50 years were associated to burnout.
Lachance et al.	2014	Canada	Survey	To assess residents' and professors' perception on the implementation of a 16-hour workday restriction	75/142 (49)	Residents and professors perceive a mild negative effect on the educational environment and quality of care, whereas their perception on QoL is opposite.
Leu et al.	2020	Australia and Switzerland	Survey	To characterize the prevalence of burnout and to identify individual and system-related predispositions for burnout	202/412 (49)	Reduced free time activities and augmented working hours increase the risk of burnout, whereas having children, salary satisfaction, and regulated weekly working hours decrease the same risk
Lund et al.	2022	USA	Survey	To investigate the relationship between gender, gendered microaggressions, and burnout	44/109 (40)	Gendered microaggressions mediate the relationship between gender and burnout
Maruscak et al.	2012	Canada	Survey	To compare the anticipated career practice patterns of surgical residents in the context of work-hour guidelines	84/274 (31)	More females plan to limit their postcall duty hours and to take a parental leave compared with their male resident colleagues.
Mayer et al.	2001	USA	Survey	To explore residents on views of parental leave and part-time practices or flexible working hours	27/71 (38)	2/3 surgeons would like to have more time off during training
Nagaraj et al.	2022	USA	Survey	To evaluate the impact of duty hours and service obligations on surgical residents' general wellbeing	(64)	Higher service obligation scores led to overall lower wellness scores regardless of the number of hours reported
Niederee et al.	2003	USA	Survey	To investigate surgical faculty and residents' attitudes towards duty-hour restrictions	3033/1314 (23)	Most residents support duty hours restrictions; surgical faculty are less supportive (more males). 70% women favor duty-hour restrictions, because of effects on personal lives
Ogawa et al.	2018	Japan	Survey	To collect data of depressive symptoms among residents	429/1241 (65)	A working week of 80 to 99.9 h was associated with a 2.83 fold higher risk and 100 h or more was associated with a 6.96-fold higher risk of developing depressive symptoms compared with a <60 h working week. No differences among sexes
Rasmussen et al.	2020	USA	Survey	To assess experiences during medical school, residency, current surgical practice and work-life balance	141/336 (42)	Female surgeons were less likely to be satisfied with their career and they report significantly more bias during their professional development and career
Seidenstein et al.	2024	USA	Survey	To assess marital status, relationship dynamics, personal life choices, the challenges and rewards of dual-healthcare relationships	56/105 (53)	Women were more likely to report at least one negative effect of a dual-healthcare relationship
Shahi et al.	2022	Nepal	Survey	To assess burnout	41/147 (28)	Being unmarried and hours of work per week (≥80 h) were significantly associated with high burnout
Tabrizian et al.	2011	USA	Survey	To investigate the reason for noncompliance with the work-hour regulation by surgical residents	54 (38)	Non-compliance with the work-hour regulation remains high and the reasons are multifactorial, as feeling guilty when leaving the hospital or told to stay late
Tawfik et al.	2021	USA	Survey	To determine factors associated with WLI	1637/4370 (37)	Largest gender disparities were observed in physicians aged 45 to 54 years, with youngest child aged ≥23 years and working <40 h per week
Troppmann et al.	2009	USA	Survey	To study career dissatisfaction and inability to achieve work-life balance	178/895 (20)	No difference among sexes for career satisfaction, but 59% believed worked too much
Von Websky et al.	2012	Switzerland	Survey	To assess trainee's satisfaction	N/A	No difference among sexes, but increasing working hours were significantly associated with dissatisfaction
Yutzie et al.	2005	USA	Survey	To gain perspective regarding general surgery career choices while examining gender differences.	5/90 (6)	More women worked 40 h per week; no difference in income for fellowship-trained surgeons, but a disparity in income favored non–fellowship-trained men
Zhang et al.	2021	USA	Survey	To assess burnout rates among breast surgeons	453/660 (69)	Breast surgeons who have been in practice for 5–9 years have particularly high overall burnout rates
Zheng et al.	2025	USA	Survey	To understand the current landscape of family planning among trainees	155/234 (66)	Work hours and female gender were associated with delayed childbirth. Females were significantly more impacted by time and career/education goals

Legend: ACGME: Accreditation Council for Graduate Medical Education; EHR: electronic health records; PD: program director; PTF: part-time clinical faculty; QoL: quality of life; WHC: work-home conflicts; WHL: workhour limitation; WLB: work-life balance; WLI: work-life imbalance.

## Results

The original search identified 3197 papers. After duplicate removal, selection for English language and exclusion for a different focus, the final inclusion for review consisted of 55 papers. The majority of the studies were surveys (*n* = 52), from the USA (*n* = 37) and of good evidence quality (Table 2).

**Table 2:** Quality of evidence in the selected papers for the systematic review according to the Newcastle–Ottawa Scale.

Author	Selection	Comparability/Exposure	Outcome	Total	Evidence quality
Antiel et al.	^⁎⁎⁎^	^⁎⁎^	^⁎⁎⁎^	8	Good
Arora et al.	^⁎⁎⁎^		^⁎⁎⁎^	6	Satisfactory
Ashrafi et al.	^⁎⁎⁎^		^⁎⁎⁎^	6	Satisfactory
Balch et al.	^⁎⁎^	^⁎⁎^	^⁎⁎⁎^	7	Good
Baldwin et al.	^⁎⁎⁎^	^⁎⁎^	^⁎⁎⁎^	8	Good
Barden et al.	^⁎⁎⁎^		^⁎⁎⁎^	6	Satisfactory
Barger et al.	^⁎⁎⁎^	^⁎⁎^	^⁎⁎⁎^	8	Good
Bendorf et al.	^⁎⁎^		^⁎⁎⁎^	5	Good
Bennett et al.	^⁎⁎^	^⁎⁎^	^⁎⁎⁎^	7	Good
Bessen et al.	^⁎⁎⁎^	^⁎⁎^	^⁎⁎^	6	Good
Bilimoria et al.	^⁎⁎⁎^		^⁎⁎⁎^	6	Satisfactory
Boerjan et al.	^⁎⁎⁎^	^⁎⁎^	^⁎⁎⁎^	8	Good
Brown et al.	^⁎⁎^	^⁎⁎^	^⁎⁎⁎^	7	Good
Chokshi et al.	^⁎⁎^	^⁎⁎^	^⁎⁎⁎^	7	Good
Coverdill et al.	^⁎⁎⁎^	^⁎⁎^	^⁎⁎⁎^	8	Good
Dahlke et al.	^⁎⁎^	^⁎⁎^	^⁎⁎⁎^	7	Good
Dholakia et al.	^⁎⁎⁎^	^⁎⁎^	^⁎⁎⁎^	8	Good
Dossa et al.	^⁎⁎⁎^	^⁎⁎⁎^	^⁎⁎^	8	Good
Dyrbye et al. 2011	^⁎⁎^	^⁎⁎^	^⁎⁎⁎^	7	Good
Dyrbye et al. 2012	^⁎⁎^	^⁎⁎^	^⁎⁎⁎^	7	Good
Elmore et al.	^⁎⁎^	^⁎⁎^	^⁎⁎⁎^	7	Good
Gifford et al.	^⁎⁎⁎^	^⁎⁎^	^⁎⁎⁎^	8	Good
Goitein et al.	^⁎⁎⁎^	^⁎⁎^	^⁎⁎⁎^	8	Good
Gray et al.	^⁎⁎⁎^	^⁎⁎^	^⁎⁎⁎^	8	Good
Hoffmann et al.	^⁎⁎⁎^		^⁎⁎⁎^	6	Satisfactory
Hughes et al. 2023	^⁎⁎⁎^	^⁎⁎⁎^	^⁎^	7	Good
Hughes et al. 2024	^⁎⁎⁎^		^⁎⁎⁎^	6	Satisfactory
Irani et al.	^⁎⁎^		^⁎⁎⁎^	5	Satisfactory
Janczewski et al.	^⁎⁎⁎^	^⁎⁎^	^⁎⁎⁎^	8	Good
Kang et al.	^⁎⁎^	^⁎⁎^	^⁎⁎⁎^	7	Good
Kawase et al.	^⁎⁎^	^⁎⁎^	^⁎⁎⁎^	7	Good
Kevric et al.	^⁎⁎^	^⁎⁎^	^⁎⁎⁎^	7	Good
Khorfan et al.	^⁎⁎⁎⁎^	^⁎⁎^	^⁎⁎⁎^	9	Very good
Kinslow et al.	^⁎⁎^	^⁎⁎^	^⁎⁎⁎^	7	Good
Kiyasu et al.	^⁎⁎⁎^		^⁎⁎⁎^	6	Satisfactory
Kuerer et al.	^⁎⁎^	^⁎⁎^	^⁎⁎⁎^	7	Good
Lachance et al.	^⁎⁎^	^⁎⁎^	^⁎⁎⁎^	7	Good
Leu et al.	^⁎⁎^	^⁎⁎^	^⁎⁎⁎^	7	Good
Lund et al.	^⁎⁎^	^⁎⁎^	^⁎⁎⁎^	7	Good
Maruscak et al.	^⁎⁎⁎^	^⁎⁎^	^⁎⁎⁎^	8	Good
Mayer et al.	^⁎⁎⁎^		^⁎⁎^	5	Satisfactory
Nagaraj et al.	^⁎⁎^	^⁎⁎^	^⁎⁎⁎^	7	Good
Niederee et al.	^⁎⁎⁎^	^⁎⁎^	^⁎⁎⁎^	8	Good
Ogawa et al.	^⁎⁎^	^⁎⁎^	^⁎⁎⁎^	7	Good
Rasmussen et al.	^⁎⁎^	^⁎⁎^	^⁎⁎⁎^	7	Good
Seidenstein et al.	^⁎⁎^		^⁎⁎⁎^	5	Satisfactory
Shahi et al.	^⁎⁎⁎^	^⁎⁎^	^⁎⁎⁎^	8	Good
Tabrizian et al.	^⁎⁎^		^⁎⁎⁎^	5	Good
Tawfik et al.	^⁎⁎⁎^	^⁎⁎^	^⁎⁎⁎^	8	Good
Troppmann et al.	^⁎⁎^	^⁎⁎^	^⁎⁎⁎^	7	Good
Von Websky et al.	^⁎⁎^	^⁎⁎^	^⁎⁎⁎^	7	Good
Yutzie et al.	^⁎⁎⁎^		^⁎⁎⁎^	6	Satisfactory
Zhang et al.	^⁎⁎^	^⁎⁎^	^⁎⁎⁎^	7	Good
Zheng et al.	^⁎⁎⁎^		^⁎⁎⁎^	6	Satisfactory

Primary themes identified in relation to an excess of working hours were: 1) burnout incidence; 2) impact of flexible policies and research time; 3) repercussion in education; 4) job satisfaction; 5) earning discrepancy between genders; 6) risk for health and wellness; 6) effects on parenthood and childcare.

1. Burnout incidence and quality of life in relation to prolonged working hours [6], [7], [8], [9], [10], [11], [12], [13], [14], [15], [16], [17], [18], [19], [20], [21], [22], [23], [24], [25]. Burnout is defined as emotional exhaustion, depersonalization, and low sense of personal accomplishment, eventually leading to attrition at work and desire to leave the job.

Burnout occur more frequently among young/mid-career surgeons aged <50 years. Prolonged working hours or ≥2 nights on call per week are consistently associated to poor work-life balance and work-home conflicts, thus surgery was not deemed a good career option any longer for up to 1 in 7 of the survey respondents.

It has been reported that women surgeons are at greater risk of family/work imbalance and burnout, with a frequent expectation that work should be prioritized over private life, a conception unfortunately also affecting the future of recruitment, as those who leave the profession look for specialties that are conducive to a more controllable lifestyle.

A general perception of a better quality of life from both genders respondents was reported with standard implementation of duty-hour restrictions; reasons to reduce working hours were more commonly associated to personal and family demands in females and to financial considerations or physical limitations in men.

2. Impact of flexible policies and research time [26], [27], [28], [29]: work hour limitations seem to affect women differently from men, as women face a more than twice decrease in time for research or other academic pursuits, and a more than three times increased sense of responsibility for supervising patient care, in comparison to male colleagues.

Furthermore, women often report exceeding working hours limitations because of administrative work, rather than proper education, with a higher electronic health record burden. Given also the need for protected time to conduct research, involvement with other responsibilities decreases the possibility to actively participate in academic projects, with female sex showing an unmet interest preventing career progression.

3. Repercussion of duty-hour restriction in education [30], [31], [32], [33]. Working hours restrictions are perceived differently by the program directors, attendings and interns, in fact more faculty than residents report warnings about a negative impact on residency education and readiness for surgical autonomy. It is interesting to report that some program directors actually favor prolonged working hours, well beyond the law-restriction. Yet, both male and female residents generally favor the working hour limitations, although in some reports females also feel more frequently unprepared for surgical training and the profession. This might be a consequence of a less supportive program or presence of bias inherent to the surgical environment, especially regarding workers adopting less than full time training.

4. Job and career satisfaction [34], [35], [36], [37], [38], [39], [40], [41], [42]: literature shows that a working week between 31 and 45 h has the highest proportion of satisfaction and that activities outside of work and sleep hours correlate highly with residents' mood, learning, and satisfaction.

Reimbursement is considered inadequate given the unpredictability and irregularity of surgeons' work hours, and women tend to be more dissatisfied with their career progression. Interestingly, breast surgeons were found to have a higher degree of job satisfaction, in consideration of the predictability of their working hours, a different setting when compared to professionals involved with emergency surgical consults during night or weekend hours.

5. Earning discrepancy between genders [43], [44], [45], [46], [47]: research shows that even when equal hours are worked, female surgeons earn less than male surgeons and have fewer opportunities to perform the most lucrative surgical procedures. Additionally, if no difference in income for fellowship-trained surgeons is observed, a disparity in income favors non–fellowship-trained men. Reasons might be various, like for example that women are more frequently underemployed, i.e. perform nursing tasks, with disrespect to their training. As a consequence, often women conceive lower salary expectations and a more negative view of salary negotiation in general.

Additionally, women frequently perform free tasks, like for example serving on committees and in administrative roles, with an increased amount of assignments, taking up time to devote to the surgical operations. With the necessity of being productive, this extra load could easily lead to an excess in working hours with no remuneration, or even worse, creating a salary gap with male colleagues.

6. Excessive working hours and risk for health and wellness [48], [49], [50], [51], [52]: fatigue resulting from long shifts is commonly described among surgeons, leading to an increased risk of avoidable errors, adverse events, and attentional failures towards patient's care in clinical activity. Additionally, fatigue has been reported also to cause illness to surgeons, not only in the body, but also in terms of mental health.

Suicidality attempts and fatal events among surgeons occur and are recognized as an unfortunate consequence of an excess of fatigue and prolonged working hours. It is important to mention that dissatisfaction with the ability to maintain healthy habits appears to be more frequently associated with female gender and junior residents, while male and senior trainees generally report themselves in a better health status. To this regard, supervisor's support appears of fundamental importance for residents and workers, in fact a higher social support in the working environment is proved to lead to a higher probability of wellness.

7. Excess of working hours and effects on parenthood and childcare [53], [54], [55], [56], [57], [58], [59]: to spend more time with family tends to be a reason for reducing working hours, especially for women and for surgeons in their 40s. Very often it is reported that the use of professional child care exceeds the number of working hours, meaning a discrepancy between the amount of hours required at work and those provided by child care.

Prolonged working hours are also known to be significantly associated with delay of childbearing, more significantly in women: trainees working over 80 h per week tend to delay childbirth more than those reporting less than 80-h workweeks.

## Discussion

The present review shows that certain aspects of the surgical profession are identifiable with those described by Goldin for greedy jobs [4]. In particular, there is evidence of a direct correlation of working beyond duty hours with burn out incidence [7], poorer health status [49], decreased productivity [60] and lower career satisfaction [61]. Literature also highlights a more detrimental effect of greedy jobs on women, being accompanied by a gender pay-gap [45] and delay in childbearing and parenthood [12].

Despite flexible policies being in place from a few years [62], there has been an unconscious bias towards those healthcare professionals who were unable to remain longer outside the agreed working hours [41], arguing it might interfere with patient's care continuity [35], [60]. Yet, as reported also in the present review, such a culture has instead a detrimental effect, leading to attentional failures during lectures, rounds, and clinical activities. Furthermore, fatigue-related preventable adverse events could result in a fatality not only for patients [48], but also for workers, thus it is not recommendable to exceed established working hours standards [20], [50].

An important finding of the present review is that women tend to be involved more frequently in administrative roles [36], leading to a potential limitation in undertaking clinical work, going to theatre and being involved in prolonged and challenging cases. Literature reports in fact that female surgeons are often underemployed in comparison to their male peers, even after accounting for subspecialty and seniority [63], with a lower number of complex procedures in their portfolio. Arguments commonly listed to explain such a difference comprehend reduced availability for competing obligations. Yet, this motivation might be seen as discriminatory, in fact on this basis only, there should not be any effect on the complexity of cases that a surgeon could perform. On the contrary, flexible time workers are often hindered in their productivity evaluation assessment and career advancement [64].

In other words, there cannot be an automatic correlation between lower availability to exceed the expected working hours, and a reduction in career advancement and seniority [40] which inevitably leads to salary and promotion gaps [36], [47]. Instead, it is necessary to determine whether, based on the circumstances of the specific case (type of operations performed, methods of performance, etc.), the proportional relationship between recognized seniority and hours of attendance at work is rational or rather constitutes discrimination against those not exceeding their working hours duty [65].

Another consideration deriving from the data reported on the present review, is that the number of hours worked and unpaid overtime are associated with poorer mental health outcomes among surgical trainees [50]; these hours are often attributable to excessive administrative work, a burden that could be avoided with the supply of technology, for example with the use of artificial intelligence to help doctors in reducing tasks not immediately requiring human interaction. To this purpose, women surgeons had a greater electronic health record load than men [36]: for instance, literature shows that residents aged <30 years are more likely to exceed duty hours to complete charting/documentation, and females more often cited guilt about leaving the hospital as to why they exceed duty hours [35], [37], [66]. These findings indeed exacerbate the culture of an “old boys club” where women are prevented from entry because of their possible commitment to family and childcare [67] and social impediments for being fully devoted to the surgical career.

This flexibility is also with regards to tenure and promotion procedures of academic faculty, in order to allow accommodation of personal and family responsibilities, while continuing career progression [36]. There is evidence in fact, that surgeons do not significantly reduce clinical productivity after maternity or other types of leaves, so childbearing should not be discouraged or delayed [68], as instead it is reported frequently in case of surgery.

Finally, a remarkable finding is that both men and women agreed that they would be more interested in a surgical career if residency programs strictly adhered to the 80-hour work week [69]; thus interventions targeted to avoid the risks of a “greedy job” would indeed appeal more aspiring candidates to enter the profession or not to leave training. This would also overcome the current lack of vocations and ensure a more sustainable recruitment, given that the quality of care and level of procedural experience could be maintained with the introduction of initiatives seeking to decrease working hours in excess, reducing burnout and improving work-life balance.

This review presents some limitations: first, literature was mainly from the USA, therefore findings might be specific to this setting and more difficult to be generalized in other healthcare contexts. Secondly, they refer mostly to the period of implementation of the 80-hours worktime directives. Third, despite the evidence of greedy job in surgery, there has been paucity of reports specifically looking at this concept, thus the need to fill the gap by a look at the current evidence.

### Recommendations

Flexibility and avoidance of “greedy” work hours are essential in surgery. The increasing use of technology, which allows for remote working, offers a key solution by reducing constraints related to space and time.

Work-commitments after the regular business hours should be discouraged, moving meetings and related issues to typical workdays. A fundamental cultural shift is needed, promoting an “anti-greed” perspective to close the gap in availability between men and women for additional work requests.

Suggested interventions for policymakers, clinicians and employers to mitigate work-home conflicts include:

• Allocating protected time: for instance, assigning two protected weekday days annually for personal matters.
• Improving the work environment: modernizing resident workspaces and providing additional meal funds [64], [70].
• Prioritizing quality over quantity: valuing the quality of time spent at work more than the sheer number of hours.
• Recognition of administrative roles in surgical careers as equally important to research and clinical tracks would ensure their contribution is better appreciated in terms of promotion and salary.
• Increasing operator interchangeability, though not strictly timing procedures, is a potential solution. This supports female surgeons in gaining equivalent operating room skills [69], fostering their contribution to the ward organization, and preventing exclusion or discrimination due to an inability to work excessive hours.

## Conclusion

Surgeons, while focusing on removing illness from patients, should protect their own rest, personal life, mental and physical health. This review highlights the impact of disregarding working hours in surgery, especially on women, leading to burnout, poor health, low productivity, decreased career satisfaction, gender pay-gap, and delayed parenthood. Respecting regular working hours directives is crucial for long-term careers and to overcome the lack of vocation to prospective surgeons. We advocate for personalized, sustainable working hours towards worker-centered care, identifying priorities for future research.

## CRediT authorship contribution statement

**Maria Irene Bellini:** Writing – original draft, Methodology, Investigation, Formal analysis, Data curation, Conceptualization. **Pasquale Passalacqua:** Writing – original draft, Formal analysis.

## Declaration of competing interest

The other author declares no conflict of interests.
